# The Risk of Adverse Birth Outcomes Among Twin Pregnancies After Influenza and Pertussis Vaccinations During Pregnancy: A Data Linkage Study

**DOI:** 10.1111/1471-0528.70156

**Published:** 2026-01-18

**Authors:** Kahlee Boyle, Sarah Graham, Michael Binks, Lisa McHugh

**Affiliations:** ^1^ School of Public Health University of Queensland Brisbane Queensland Australia; ^2^ Menzies School of Health Research Charles Darwin University Darwin Northern Territory Australia; ^3^ Women and Kids Theme South Australian Health and Medical Research Institute Adelaide South Australia Australia

**Keywords:** influenza, maternal vaccination, multiple pregnancy, pertussis, safety, twins

## Abstract

**Objective:**

To compare risks of adverse birth outcomes between maternally vaccinated and unvaccinated twin pregnancies.

**Design:**

Multi‐jurisdictional data linkage cohort study.

**Setting:**

All registered births in Queensland (Qld) and Northern Territory (NT), Australia between 1 January 2012 and 31 December 2017.

**Population:**

Twin pregnancies with infants born ≥ 20 weeks gestation and weighing ≥ 400 g.

**Methods:**

We used Cox proportional‐hazard models to calculate risk, with maternal vaccination status as the time‐varying exposure, and national birthweight percentile charts specific for Australian‐born twins to accurately reflect risk among small for gestational age (SGA) infants.

**Main Outcome Measures:**

Adverse birth outcomes including preterm birth, stillbirth and SGA infants.

**Results:**

Among our cohort of *n* = 11 435 infants, there was no statistically significant increased risk of preterm births, stillbirths or SGA infants between women who received a maternal influenza or pertussis vaccination and unvaccinated women. We observed a non‐significant lower risk of SGA infants among pertussis vaccinated Qld (aHR 0.85, 95% CI 0.70–1.03) and NT women (aHR 0.50, 95% CI 0.17–1.44), and a lower risk of preterm infants among influenza vaccinated Qld women (aHR 0.93, 95% CI 0.84–1.03) and pertussis vaccinated NT women (aHR 0.78, 95% CI 0.44–1.38).

**Conclusion:**

Maternal influenza and pertussis vaccinations did not increase the risk of preterm birth, stillbirth or SGA infants among twin pregnancies. These novel and important findings alleviate safety concerns for a cohort that carries a higher baseline risk of adverse birth outcomes compared to singleton pregnancies.

## 1 Introduction

Influenza and pertussis are two of the most frequently reported vaccine preventable diseases in Australia [1]. Pregnant women are at a higher risk of severe illness and even death from respiratory viruses, including influenza [2, 3], and infants aged < 6 months that are too young to receive direct immunisation are also highly vulnerable. Infants are disproportionately susceptible to severe influenza and pertussis infections due to their immature immune systems [4, 5]. Passive protection of infants by transplacental transfer of antibodies from the maternally vaccinated mother to the foetus is the best strategy until infants are old enough to receive direct vaccination; for pertussis this is multiple doses throughout infancy from 6 weeks of age, and at least one dose from 6 months of age for influenza.

Australia has fully‐funded programs for both seasonal inactivated influenza vaccination (IIV) and pertussis‐containing vaccines (dTpa) for pregnancy under the National Immunisation Program [6]. Despite this funding, maternal vaccination rates remain suboptimal across Australia, particularly among First Nations women [7]. Safety concerns are often cited as a prominent barrier to maternal vaccination uptake globally [8]. Studies on singleton pregnancies have provided reassuring evidence regarding the safety and effectiveness of maternal vaccinations [8, 9], but no Australian study has examined the uptake or safety of IIV and/or dTpa vaccinations among women carrying twins, triplets or higher orders (multiple pregnancy).

Compared to singleton pregnancies, multiples pose a higher risk of adverse birth outcomes, including preterm births, stillbirths and small for gestational age (SGA) infants [10]. In Australia, the stillbirth rate among twin pregnancies is over three times the rate observed in singleton pregnancies, and nearly 65% of twin births occur prematurely compared to 6.6% of singleton births [11, 12]. SGA and preterm infants are at a particularly higher risk of experiencing serious complications from respiratory infections compared to other infants [13]. This increased risk of severe respiratory illness in conjunction with the higher prevalence of adverse birth outcomes indicates a need to increase maternal vaccination uptake among multiple pregnancies. This study aimed to examine whether there was an increased risk of an adverse birth outcome among women carrying a multiple pregnancy who were vaccinated with influenza or pertussis during pregnancy compared to unvaccinated women carrying a multiple pregnancy.

## 2 Methods

### 2.1 Study Design and Participants

We conducted a secondary analysis of Queensland (Qld) and Northern Territory (NT) linked administrative health datasets, previously described elsewhere [14]. Women with a multiple pregnancy (two or more foetuses in the same pregnancy) who birthed in Qld or the NT, Australia between 1 January 2012 and 31 December 2017 were included. Consistent with the nationally recognised criteria for a registerable infant birth or death, infants born prior to 20^+0^ weeks gestation and/or weighing less than 400 g were excluded.

Women who had more than one multiple pregnancy during the study period were only captured once in the final analysis using the first multiple pregnancy occurrence. Due to small numbers and the potential to impact privacy and confidentiality, all triplet and higher order multiples were excluded from the final analysis.

Funded dTpa vaccinations were introduced and recommended for pregnant women in their third trimester from 2014 in Qld and 2015 in the NT. Accordingly, the dTpa models were restricted to births occurring from these respective years until 2017. IIV was fully funded and recommended in Australia from 2010 [6], therefore, IIV models included births from 2012 to 2017.

### 2.2 Exposure

The exposure of interest was maternal vaccination with IIV/dTpa, relevant to the model, administered at any gestation of pregnancy. The gestation in weeks at time of vaccination was derived from the date of vaccination and gestational age at birth.

### 2.3 Outcomes

The birth outcomes of interest were preterm birth, stillbirth and SGA infants. Preterm birth was defined as birth occurring between 20^+0^ and 36^+6^ weeks gestation. Stillbirth was defined as a foetal death at 20^+0^ or more completed weeks gestation and/or weighing 400 g or more. Infants less than the 10th percentile of birthweight for gestational age and sex were considered SGA. Our study classified SGA infants according to the 2015 male and female national birthweight percentile charts for twins born in Australia [15].

### 2.4 Covariates

Models were adjusted for covariates including maternal age, remoteness of maternal residence (Accessibility/Remoteness Index of Australia), parity, smoking status during pregnancy and socio‐economic indexing (Index of Relative Advantage and Disadvantage, Australian Bureau of Statistics Socio‐Economic Indexes for Areas [SEIFA] 2011 [16]). We also considered seasonality a confounder for IIV only, as although adverse birth outcomes are seasonal [17], dTpa vaccination during pregnancy is not. Despite ongoing social and health inequities faced by First Nations peoples in Australia, maternal First Nations identity is not considered an independent risk factor and therefore has not been included in our study as a covariate. Sensitivity analyses using E‐values were conducted to assess the potential impact of unmeasured confounding.

### 2.5 Statistical Analysis

Unadjusted risk ratios with 95% confidence intervals (95% CI) and counts with corresponding proportions were calculated for the demographic, pregnancy and health characteristics of the cohort by jurisdiction and vaccination status. Cox proportional‐hazard models were used to estimate hazard ratios (HR) and adjusted hazard ratios (aHR) for each primary outcome between vaccinated and unvaccinated women. Separate models were estimated for (i) IIV and dTpa vaccinations; (ii) preterm birth, stillbirth and SGA infants; and (iii) Qld and NT to account for jurisdictional demographic differences. To address immortal time bias, vaccinated women contributed unvaccinated and vaccinated time‐at‐risk by using gestation at vaccination as a time‐varying exposure. In Australia, births at < 20 weeks gestation are classified as a miscarriage (spontaneous abortion), therefore time‐at‐risk for all outcomes commenced at 20 weeks gestation. Infants born without the outcome of interest were censored at the gestation (week) of birth, or when time‐at‐risk ended (37 weeks gestation for preterm birth; gestation of stillbirth for preterm birth and SGA). To ensure robustness, we compared estimates from the primary analyses against separate Fine‐Grey models to assess the impact of stillbirth as a competing risk for preterm birth and SGA. HRs for preterm birth were calculated at the pregnancy level as all twin infant pairs were born at the same gestation. HRs for stillbirth and SGA were calculated at the individual infant level, with robust standard errors to account for the intra‐pregnancy clustering between twin infant pairs. Results were reported as HRs with 95% CI, and we considered 95% CI that did not cross one and *p* values < 0.05 as statistically significant.

We used multiple imputation by chained equations (MICE) with the addition of ten datasets (combined according to Rubin's rules) in all Cox models to avoid complete‐case analysis bias for missing smoking status data (Qld < 1%; NT 8.5%). All exposure and outcome variables, and covariates were included in the MICE models. The proportional hazards assumption was assessed using a test of Schoenfeld residuals, including on randomly extracted datasets for the MICE data. We satisfied the proportional hazards assumption for preterm birth (Qld) using stratified Cox models with a separate stratum for preterm birth category (extreme preterm, very preterm, moderate to late preterm). For the outcome SGA, IIV (NT) was stratified by season of conception, and dTpa (Qld) was stratified by parity with a separate stratum for primiparous, one prior birth and two or more prior births. Data were analysed using Stata statistical software package v.19 (StataCorp, College Station, Texas).

## 3 Results

We excluded 171 infants who did not meet the criteria of a registerable birth or death (Figure S1). Characteristics of the excluded triplet and higher order pregnancies and subsequent sets of multiples were summarised (Table S1).

There were 11 435 infants born from 5757 twin pregnancies in Qld and the NT between 1 January 2012 and 31 December 2017. Qld twin pregnancies accounted for 95% of the cohort. Baseline demographic and pregnancy characteristics were summarised by vaccination status (Table 1) and jurisdiction (Table S2). We observed statistically significant differences between the Qld and NT populations, including maternal age, maternal First Nations status, remote area of residence, smoking status during pregnancy, and pregnancy‐related and pre‐existing medical conditions.

**TABLE 1:** Demographic, pregnancy and health characteristics by influenza (IIV) and pertussis (dTpa) vaccination status (after the introduction of dTpa), twin births Queensland and Northern Territory 2012–2017.

	Total pregnancies	Unvaccinated	Vaccinated (IIV or dTpa)	IIV	dTpa	Both IIV and dTpa
Characteristic (*N*%)	*n* = 5757	*n* = 4171 (72%)	*n* = 1586 (28%)	*n* = 140 (2%)	*n* = 894 (16%)	*n* = 552 (10%)
**Jurisdiction**						
Northern Territory	283 (5)	238 (6)	45 (3)	16 (11)	17 (2)	12 (2)
Queensland	5474 (95)	3933 (94)	1541 (97)	124 (89)	877 (98)	540 (98)
**Maternal age group at infant birth**						
< 20 years	106 (2)	93 (2)	13 (1)	< 5 (1)	9 (1)	< 5 (1)
20–34 years	4098 (71)	2967 (71)	1131 (71)	103 (74)	627 (70)	401 (73)
≥ 35 years	1553 (27)	1111 (27)	442 (28)	36 (26)	258 (29)	148 (27)
**Body Mass Index** ^a^, ^b^						
Underweight	259 (5)	195 (5)	65 (4)	9 (7)	39 (5)	16 (3)
Healthy	2655 (50)	1897 (50)	758 (50)	61 (50)	427 (50)	270 (51)
Overweight	1298 (24)	931 (24)	367 (24)	25 (20)	195 (23)	147 (28)
Obese	1113 (21)	793 (21)	320 (21)	28 (23)	195 (23)	97 (18)
**SEIFA Index Score**						
Index 1–3	1628 (28)	1270 (30)	358 (23)	39 (28)	205 (23)	114 (21)
Index 4–6	1634 (28)	1153 (28)	481 (30)	44 (31)	287 (32)	150 (27)
Index 7–10	2495 (43)	1748 (42)	747 (47)	57 (41)	402 (45)	288 (52)
**Parity**						
0 prior births (primiparous)	2361 (41)	1645 (39)	716 (45)	49 (35)	401 (45)	266 (48)
1 prior birth	1821 (32)	1341 (32)	490 (31)	43 (31)	281 (31)	166 (30)
**≥** 2 prior births	1565 (27)	1185 (28)	380 (24)	48 (34)	212 (24)	120 (22)
**Other**						
First Nations	370 (6)	291 (7)	79 (5)	15 (11)	38 (4)	26 (5)
Australian born	4773 (83)	3465 (83)	1308 (82)	118 (84)	750 (84)	440 (80)
Remote area of residence	217 (4)	168 (4)	49 (3)	11 (8)	25 (3)	13 (2)
Antenatal care in 1st trimester	4396 (78)	3042 (75)	1354 (86)	116 (84)	754 (85)	484 (88)
Public hospital birth^a^	2937 (54)	2161 (55)	776 (50)	68 (55)	483 (55)	225 (42)
Smoked during pregnancy	668 (12)	541 (13)	127 (8)	21 (15)	71 (8)	35 (6)
Caesarean section birth	4053 (70)	2901 (70)	1152 (73)	99 (71)	664 (74)	389 (70)
Pregnancy‐related condition^c^	1117 (19)	772 (19)	345 (22)	29 (21)	195 (22)	121 (22)
Pre‐existing medical condition^d^	974 (17)	675 (16)	299 (19)	22 (16)	169 (19)	108 (20)

*Note:* Denominators reflect number of unique pregnancies and differ due to missing data. Pertussis vaccine (dTpa) *N*(%) from 2014 in Qld and 2015 in NT.

^a^ Qld data only.

^b^ Derived from body mass index variable using Australian categories.

^c^ Derived variable: presence of one or more of gestational diabetes, gestational hypertension, preeclampsia, antepartum haemorrhage.

^d^ Derived variable: presence of one or more of asthma, hypertension, diabetes (type I or II), anaemia, cardiac condition, renal condition.

Most women were unvaccinated (72%); 2% received only IIV, 16% received only dTpa (from 2014 in Qld; 2015 in NT), and 10% received both IIV and dTpa. Other vaccination characteristics are presented in Table S3.

### 3.1 Birth Outcomes

#### 3.1.1 Stillbirth

In Qld, 147 infants (1.4%) were stillborn between 2012 and 2017, with 102 stillbirths occurring between 2014 and 2017. The mean gestational age of stillborn infants was 28 weeks (range 20–40 weeks). In the NT, nine infants (1.6%) were stillborn between 2012 and 2017 with four stillbirths occurring after the introduction of dTpa in 2015. NT stillbirths occurred between 21 and 37 weeks gestation (mean 26 weeks). The median time between vaccination and stillbirth was 13 weeks (range 3–24 weeks) for IIV and 6.5 weeks (range 1–11 weeks) for dTpa, noting 89% of dTpa was administered in the third trimester. There were no stillbirths among vaccinated NT women during the study period; therefore, we were unable to model the risk of stillbirth following IIV or dTpa vaccination. Due to the small proportion of stillbirths, estimates remained similar when modelling stillbirth as a competing risk (Table S4).

#### 3.1.2 Small for Gestational Age

In Qld, 917 infants (8%) were SGA (*n* = 579 between 2014 and 2017), and in the NT 56 infants (10%) (*n* = 28 between 2015 and 2017) were SGA. The median time between IIV administration and the birth of a SGA infant was 11 weeks (range 2–33 weeks), and 5 weeks (range 0–25 weeks) for dTpa.

#### 3.1.3 Preterm Birth

In Qld, 3639 births (66%) were preterm, comprising 3054 (84%) moderate to late preterm, 372 (10%) very preterm and 213 (6%) extremely preterm. In the NT, 178 births (63%) occurred preterm comprising 146 (82%) moderate to late preterm, 19 (11%) very preterm and 13 (7%) extremely preterm. The median time between IIV administration and preterm birth was 11 weeks (range 1–35 weeks), and 5 weeks (range 0–36 weeks) for dTpa vaccination.

### 3.2 Influenza Vaccination Outcomes

#### 3.2.1 Main Findings

**FIGURE 1:**
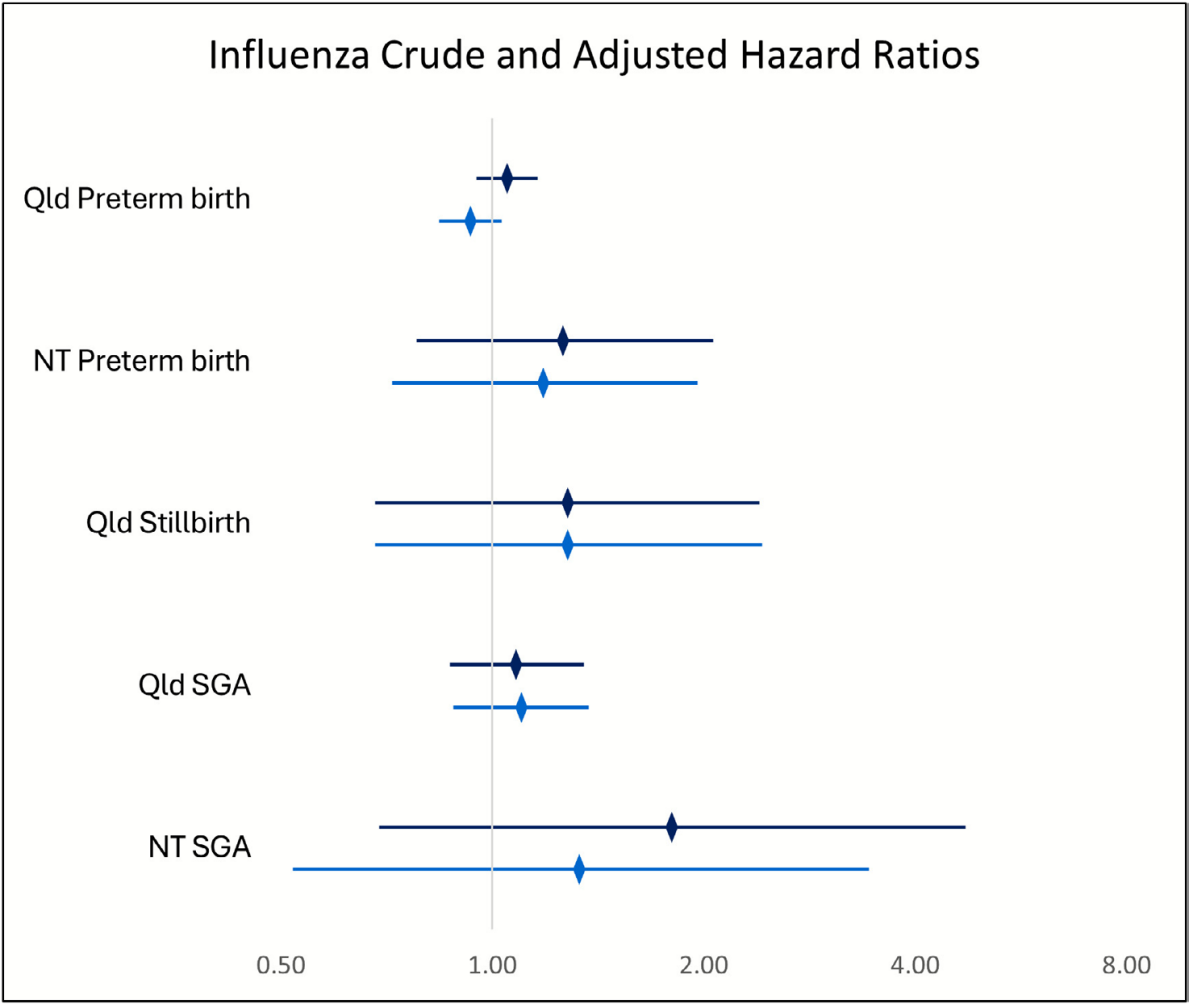
Influenza crude and adjusted hazard ratios, all jurisdictions and birth outcomes, 2012–2017.

Compared with unvaccinated women, the absolute risk among IIV vaccinated women was lower for stillbirth (Qld 1.4% vs. 1.1%; NT 1.8% vs. 0%), marginally higher for preterm birth (Qld 66.4% vs. 67.1%; NT 62.5% vs. 63.6%), and higher for SGA (Qld 8.1% vs. 8.4%; NT 9.0% vs. 14.6%) (Table S5). Among Qld women, we found no statistically significant difference in the risk of stillbirth between IIV vaccinated and unvaccinated women before (HR 1.28, 95% CI 0.68–2.40) or after adjusting for covariates (aHR 1.28, 95% CI 0.68–2.42) (Table 2). There was no statistically significant difference in the risk of SGA infants between IIV vaccinated and unvaccinated pregnancies in Qld before (HR 1.08, 95% CI 0.87–1.35) or after adjustment for covariates (aHR 1.10, 95% CI 0.88–1.37) (Table 2). Similarly, in the NT, crude (HR 1.80, 95% CI 0.69–4.72) and adjusted (aHR 1.33, 95% CI 0.52–3.44) analysis demonstrated no difference in the risk of SGA infants between vaccinated and unvaccinated women (Table 2). In Qld, the association between IIV and preterm birth indicated a lower risk following adjustment (aHR 0.93, 95% CI 0.84–1.03) in comparison to the higher risk observed in the crude analysis (HR 1.05, 95% CI 0.95–1.16); however, this remained non‐significant (Table 2). Likewise, in the NT, there was no increased risk in preterm birth among women who received IIV in comparison to unvaccinated women before (HR 1.26, 95% CI 0.78–2.06) or after adjusting for covariates (aHR 1.18, 95% CI 0.72–1.96) (Table 2). A comparison of crude and adjusted HRs is provided in Figure 1.

**TABLE 2:** Birth outcomes by vaccination status; crude and adjusted Cox proportional hazard ratios for birth outcomes by maternal vaccination status, twin births Queensland and Northern Territory between 2012 and 2017.

	Total *N* (%)	Unvaccinated *N* (%)	Vaccinated *N* (%)	Crude HR (95% CI)	*p*	Adjusted HR^a^ (95% CI)	*p*
**Influenza**							
Queensland							
Stillbirth^b^	147 (1)	132 (90)	15 (10)	1.28 (0.68–2.40)	0.447	1.28 (0.68–2.42)	0.446
SGA^b^, ^d^	917 (8)	806 (88)	111 (12)	1.08 (0.87–1.35)	0.478	1.10 (0.88–1.37)	0.391
Preterm birth^c^, ^d^	3639 (66)	3194 (88)	445 (12)	1.05 (0.95–1.16)	0.372	0.93 (0.84–1.03)	0.184
Northern Territory							
Stillbirth^b^	9 (2)	9 (100)	< 5 (0)	NA	NA	NA	NA
SGA^b^, ^d^	56 (10)	48 (86)	8 (14)	1.80 (0.69–4.72)	0.232	1.33 (0.52–3.44)	0.550
Preterm birth^c^, ^d^	178 (63)	160 (90)	18 (10)	1.26 (0.78–2.06)	0.349	1.18 (0.72–1.96)	0.512
**Pertussis** ^e^							
Queensland							
Stillbirth^b^	102 (1)	82 (80)	20 (20)	1.15 (0.61–2.18)	0.660	1.14 (0.60–2.15)	0.691
SGA^b^, ^d^	579 (8)	381 (66)	198 (34)	0.89 (0.73–1.08)	0.230	0.85 (0.70–1.03)	0.090
Preterm birth^c^, ^d^	2382 (67)	1477 (62)	905 (38)	1.10 (1.01–1.20)	0.028	1.02 (0.93–1.11)	0.656
Northern territory							
Stillbirth^b^	4 (1)	4 (100)	< 5 (0)	NA	NA	NA	NA
SGA^b^, ^d^	28 (9)	25 (89)	3 (11)	0.52 (0.17–1.60)	0.254	0.50 (0.17–1.44)	0.201
Preterm birth^c^, ^d^	95 (64)	80 (84)	15 (16)	0.83 (0.47–1.45)	0.507	0.78 (0.44–1.38)	0.402

^a^ Adjusted for maternal age, remote residence, SEIFA Index Score, parity, smoking status during pregnancy, season of conception (IIV only). SGA NT stratified (season of conception), SGA Qld stratified (parity [0, 1, ≥ 2]).

^b^ Stillbirth and SGA (infant outcome) clustered (same mother for each infant pair).

^c^ Preterm birth (pregnancy outcome), births from 37 weeks censored, Qld IIV/dTpa stratified by preterm birth category.

^d^ Stillbirths censored at gestational age for SGA and preterm birth outcomes.

^e^ Pertussis (dTpa) recommended during the third trimester of pregnancy from 2014 in Qld and 2015 in NT. Total cohort includes births from 2014 (Qld)/2015 (NT) to 2017.

#### 3.2.2 Other Findings

In Qld, women residing in SEIFA index areas 1–3, indicating an area of greater disadvantage on a 1–10 index, had a 72% higher risk of stillbirth compared to women residing in more advantaged areas (SEIFA index area 4–6) (aHR 1.72, 95% CI 1.02–2.89) (Table S6). We observed Qld and NT women who smoked during pregnancy had a higher risk of SGA infants (Qld aHR 2.44, 95% CI 2.01–2.96; NT aHR 2.04, 95% CI 1.14–3.67) (Table S7). Furthermore, Qld smokers had a higher risk of preterm birth (aHR 1.13, 95% CI 1.02–1.26) (Table S8). Qld women aged ≥ 35 years had a higher risk of SGA infants (aHR 1.30, 95% CI 1.11–1.52). Conversely, Qld women demonstrated a 20% lower risk of a SGA infant with each increasing previous birth (aHR 0.80, 95% CI 0.74–0.86). Remote living NT women experienced a higher risk of SGA infants (aHR 2.19, 95% CI 1.03–4.66) (Table S7).

### 3.3 Pertussis Vaccination Outcomes

#### 3.3.1 Main Findings

**FIGURE 2:**
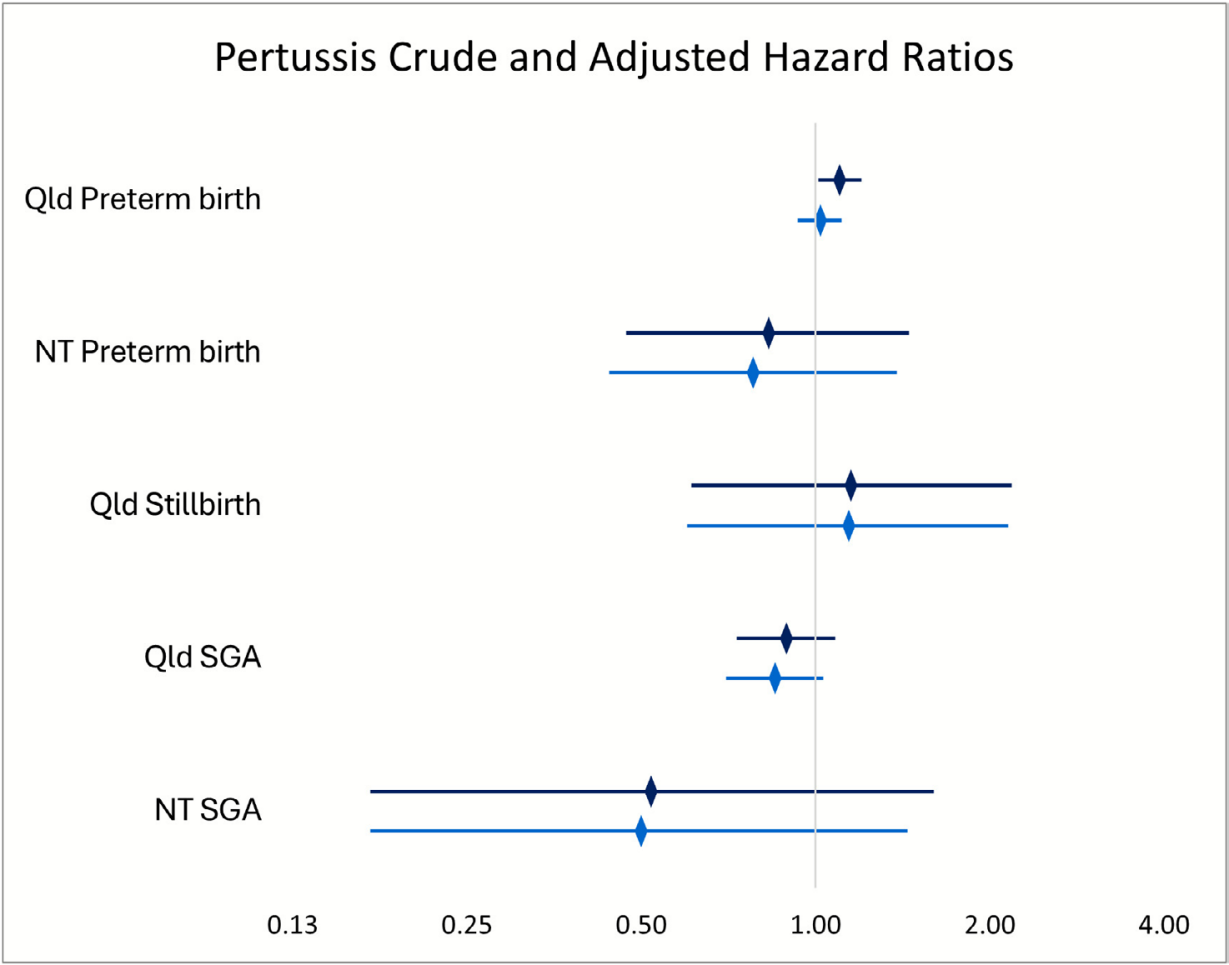
Pertussis crude and adjusted hazard ratios, all jurisdictions and birth outcomes, 2014 (Qld) 2015 (NT)—2017.

Absolute risk was lower among dTpa vaccinated women across all outcomes: stillbirth (Qld 1.6% vs. 0.7%; NT 1.8% vs. 0%), SGA (Qld 8.6% vs. 7%; NT 10% vs. 6.3%), and preterm birth (Qld 67.4% vs. 64%; NT 63.5% vs. 56.3%) (Table S5). Among Qld pregnancies, there was no statistically significant difference in the risk of stillbirth between dTpa vaccinated and unvaccinated women before (HR 1.15, 95% CI 0.61–2.18) or after adjusting for covariates (aHR 1.14, 95% CI 0.60–2.15) (Table 2, Table S9). Women who received dTpa vaccination had a lower risk of SGA infants; however, these findings were not significant in the crude (Qld HR 0.89, 95% CI 0.73–1.08; NT HR 0.52, 95% CI 0.17–1.60) or adjusted models (Qld aHR 0.85, 0.70–1.03; NT aHR 0.50, 95% CI 0.17–1.44) (Table 2). There was a significantly higher risk of preterm birth among Qld women who received dTpa in pregnancy (HR 1.10, 95% CI 1.01–1.20); however, this did not remain after adjusting for covariates (aHR 1.02, 95% CI 0.93–1.11) (Table 2). In the NT, we observed a lower risk of preterm birth for women who received dTpa compared to unvaccinated women, which remained non‐significant before (HR 0.83, 95% CI 0.47–1.45) and after adjustment (aHR 0.78, 95% CI 0.44–1.38) (Table 2). A comparison of crude and adjusted HRs is provided in Figure 2.

#### 3.3.2 Other Findings

The adjusted models demonstrated Qld smokers had a significantly higher risk of SGA infants (aHR 2.33, 95% CI 1.82–2.97) (Table S10). We observed a significantly lower risk of preterm birth for Qld women with each increasing previous birth (aHR 0.95, 95% CI 0.92–0.99), and for NT women aged ≥ 35 years (aHR 0.51, 95% CI 0.28–0.93) (Table S11).

Kaplan–Meier plots are presented in the Supporting Information (Figures S2–S11). Findings were robust against unmeasured confounding, with E‐values ranging 1.16–3.41 (Table S12); the lower E‐value (1.16) is attributable to an aHR close to the null.

## 4 Discussion

### 4.1 Main Findings

Between 2012 and 2017, there was demonstrably poor uptake of both recommended nil‐cost maternal vaccinations among twin pregnancies in Qld and the NT. We found no statistically significant increased risk of preterm births, stillbirths or SGA infants between women who received IIV or dTpa vaccination in pregnancy and unvaccinated women carrying a twin pregnancy in Qld or the NT.

### 4.2 Strengths and Limitations

This is the first study to investigate the association between maternal vaccinations and adverse birth outcomes among twin pregnancies. Given the substantial contribution of multiples towards overall perinatal morbidity and mortality [15, 18], our study fills a key gap in maternal vaccination safety research. This study has several strengths, notably, complete Qld and NT population data of all registered births over six consecutive years, and the use of gold standard time‐to‐event modelling to limit immortal time bias. Immortal time refers to the period of time the outcome could not occur as a result of the exposure, and in maternal vaccination studies this is the time between conception and vaccination [19]. Unless ‘time‐to‐event’ data analysis methods are used, the risk of an adverse birth outcome is underestimated in the maternally vaccinated [20, 21]. A further strength of this study is the use of Australian twin‐specific birthweight percentile charts to classify SGA infants. Singletons and twins remain on a similar growth trajectory until approximately 26–30 weeks gestation at which time twin growth reduces [22, 23, 24]. Over‐classification of SGA twins occurs frequently when using singleton birthweight percentile charts which can result in reduced accuracy in identifying infants at actual risk of SGA‐related adverse neonatal outcomes [25].

Our study also had limitations, specifically access to data on potential risk factors such as whether birth was spontaneous or iatrogenic, and chorionicity. In twin pregnancies, chorionicity plays a fundamental part in assessing the risk of adverse outcomes and guiding whether an iatrogenic preterm birth is recommended to prevent adverse outcomes, particularly stillbirth related to twin specific conditions such as twin‐to‐twin transfusion syndrome [13, 26, 27, 28, 29]. However, the robust methodologies we have used for our data analysis modelling and outcome comparisons show that our study population characteristics are generalisable to the wider population.

### 4.3 Interpretation

Healthcare provider recommendations have an integral role in influencing the acceptance of IIV and dTpa vaccinations during pregnancy [8]. As previous studies have only examined the safety of maternal vaccinations among singleton pregnancies, the results from our study provide reassurance that, similarly to singleton pregnancies, maternally vaccinated women carrying a twin pregnancy are not at an increased risk of experiencing the adverse birth outcomes presented here [9]. This assurance is crucial as twin pregnancies experience higher baseline rates of preterm births, stillbirths and SGA infants, thereby increasing the infants' potential for severe morbidity and mortality from pertussis and influenza infections. Furthermore, our findings likely carry clinical significance at a population level. Given the high absolute risk of preterm birth among twin pregnancies—66% in this study which is generalisable at 65% nationally—the 7% lower risk observed among (Qld) IIV vaccinated women may be clinically important. Similarly, the 15% lower risk of SGA infants among (Qld) women who received a dTpa vaccination, compared to unvaccinated women, represents a clinically meaningful reduction among twin pregnancies.

Our findings within the influenza models demonstrated NT vaccinated women had a higher risk of preterm and SGA infants than observed within the Qld study population. Despite being non‐significant, IIV vaccinated women in Qld had a 7% lower risk of preterm birth compared to a 9% higher risk in vaccinated NT women. Furthermore, whilst we did not observe any stillbirths among vaccinated NT women, Australian birthing statistics show NT women experience stillbirths at a rate double that of other jurisdictions [30]. A 2011 study on comparative high‐income countries such as Canada, New Zealand and the United States found their First Nations women had higher odds of preterm birth and stillbirth in comparison to non‐First Nations women [31]. Considering the higher proportion of First Nations women birthing in the NT (30%) compared to Qld (5%), it is plausible our findings directly reflect the population demographics. Therefore, we must consider that if these non‐significant results are real and are being driven by the higher proportion of First Nations women in the NT, then the numerical difference is clinically important. First Nations women and their infants are at an increased risk of severe health complications, hospitalisations, intensive care unit admissions and death from influenza and pertussis infections, yet we found the administration of maternal vaccinations among this priority group remained substantially lower than their non‐First Nations counterparts [7, 32]. This suggests that more effort is required to co‐design culturally appropriate, inclusive maternal vaccination programs to increase the administration of recommended vaccinations among First Nations women.

In contrast, despite the difference in rates of smokers in the NT (24%) compared to Qld (11%), smoking during pregnancy was observed as a statistically significant risk factor for SGA in three models, with the fourth model (NT pertussis) approaching statistical significance. Our findings are consistent with earlier non‐vaccination related studies which demonstrate that smoking during pregnancy is associated with an increased risk of SGA [33, 34]. In addition to the potential impacts on the immune response following vaccination, smokers are also more susceptible to respiratory infections [35, 36]. However, despite these risks, we identified smokers had considerably lower uptake of maternal vaccinations. By virtue of being a health behaviour, cessation of smoking during pregnancy is one of the few risk factors that can be changed to improve maternal and infant outcomes. In light of our reassuring findings on the safety of maternal vaccinations, it is important that healthcare providers continue to promote public health messaging on maternal smoking and give all women evidence‐based information and recommendations to receive IIV and dTpa vaccinations.

Furthermore, whilst our results and other literature demonstrate that women who have not birthed previously are more likely to be maternally vaccinated than multiparous women [21, 37], we did observe a lower risk of preterm and SGA infants for each increasing prior birth in some models. Retaining the uptake of maternal vaccinations once a woman has birthed previously, and indeed continuing to increase uptake among primiparous women, is a foreseeable challenge. Vaccine confidence has been severely impacted in recent years, fuelled by misinformation spread throughout the COVID‐19 pandemic [38]. Our important safety evidence needs to be disseminated to the wider community in a clear and transparent way to regain vaccine confidence and increase vaccine uptake during pregnancy.

## 5 Conclusion

Maternal vaccination provides passive immunity to infants too young to receive direct vaccination, with the additional benefit of protecting pregnant women. Women carrying a twin pregnancy and their infants have not previously been included in maternal vaccine safety studies despite their increased risk of adverse birth outcomes and potential for increased morbidity and mortality from respiratory infections. Our study has provided novel and critical evidence to fill this gap and importantly give healthcare providers and pregnant individuals reassurance that maternal IIV and dTpa vaccinations do not increase their risk of preterm birth, stillbirth or SGA infants. Nationally, multiple births only make up a small proportion of all births, but the adverse perinatal outcomes are disproportional. We must do better to increase maternal vaccination uptake.

## Author Contributions

K.B. contributed to the study concept and design, performed the statistical analysis, interpreted the data, and contributed to the writing of the manuscript. S.G. contributed to the statistical analysis and writing of the manuscript. M.B. contributed to the acquisition of the data and writing of the manuscript. L.M. contributed to the acquisition of the data, developed the study concept and design, contributed to the interpretation of the data and writing of the manuscript.

## Funding

This study was originally funded by a National Health and Medical Research Council (NHMRC) Project Grant (GNT1141510). LM is supported by an NHMRC Emerging Leader (EL) 1 Investigator Grant (GNT2016407) at the University of Queensland. The funding sources had no role in the planning, study design, data collection, analysis or interpretation, reporting or publication of this study.

## Ethics Statement

Multi‐jurisdictional ethics committee approvals were gained from the University of Queensland, Queensland Health and Royal Brisbane and Women's Hospital (HREC/2018/QRBW/47660), and Menzies School of Health Research (HREC 2018–3199). As this was a retrospective study of deidentified, linked data, patients could not be involved in the design, recruitment, or conduct of the study.

## Conflicts of Interest

The authors declare no conflicts of interest.

## Supporting information


**Figure S1:** Flow diagram of participants by jurisdiction and vaccination status (influenza and post‐pertussis), 2012–2017.
**Figure S2:** KM Plot Influenza Qld, preterm birth, by vaccination status.
**Figure S3:** KM Plot Influenza NT, preterm birth, by vaccination status.
**Figure S4:** KM Plot Influenza Qld, SGA, by vaccination status.
**Figure S5:** KM Plot Influenza NT, SGA, by vaccination status.
**Figure S6:** KM Plot Influenza Qld, stillbirth, by vaccination status.
**Figure S7:** KM Plot Pertussis Qld, preterm birth, by vaccination status.
**Figure S8:** KM Plot Pertussis NT, preterm birth, by vaccination status.
**Figure S9:** KM Plot Pertussis Qld, SGA, by vaccination status.
**Figure S10:** KM Plot Pertussis NT, SGA, by vaccination status.
**Figure S11:** KM Plot Pertussis Qld, Stillbirth, by vaccination status.

**Table S1:** Demographic, pregnancy and health characteristics—excluded pregnancies, multiple births 2012–2017.
**Table S2:** Demographic, pregnancy and health characteristics, by jurisdiction, twin births 2012–2017.
**Table S3:** Demographic, pregnancy and health characteristics unadjusted risk ratios, by jurisdiction and vaccination status, twin births influenza (2012) and post‐pertussis (Queensland 2014, Northern Territory 2015)—2017.
**Table S4:** Comparison of crude and adjusted estimates using Cox‐proportional hazard and competing risks (Fine‐Grey) models with stillbirth a competing risk for preterm birth and small for gestational age.
**Table S5:** Absolute risk of small for gestational age, stillbirth and preterm birth—overall, by jurisdiction and vaccination status.
**Table S6:** Crude and adjusted Cox proportional hazard ratios for stillbirth and maternal influenza vaccination, Queensland twin births 2012–2017.
**Table S7:** Crude and adjusted Cox proportional hazard ratios for small for gestational age and maternal influenza vaccination, Queensland and Northern Territory twin births 2012–2017.
**Table S8:** Crude and adjusted Cox proportional hazard ratios for preterm birth and maternal influenza vaccination, Queensland and Northern Territory twin births 2012–2017.
**Table S9:** Crude and adjusted Cox proportional hazard ratios for stillbirth and maternal pertussis vaccination, Queensland twin births 2014–2017.
**Table S10:** Crude and adjusted Cox proportional hazard ratios for small for gestational age and maternal pertussis vaccination, Queensland (2014) and Northern Territory (2015)—2017 twin births.
**Table S11:** Crude and adjusted Cox proportional hazard ratios for preterm birth and maternal pertussis vaccination, Queensland (2014) and Northern Territory (2015)—2017 twin births.
**Table S12:** Sensitivity analyses to assess unmeasured confounding (E‐value).

## Data Availability

The data that support the findings of this study are available on request from the corresponding author. The data are not publicly available due to privacy or ethical restrictions.
