# Supplementary material for: The Risk of Adverse Birth Outcomes Among Twin Pregnancies After Influenza and Pertussis Vaccinations During Pregnancy: A Data Linkage Study
Source: BJOG. 2026 Jan 18;133(5):1083–92. doi: 10.1111/1471-0528.70156 (PMC12972857; doi:10.1111/1471-0528.70156)
Supplement: Supplementary file 1 — Figure S1: Flow diagram of participants by jurisdiction and vaccination status (influenza and post‐pertussis), 2012–2017. Figure S2: KM Plot Influenza Qld, preterm birth, by vaccination status. Figure S3: KM Plot Influenza NT, preterm birth, by vaccination status. Figure S4: KM Plot Influenza Qld, SGA, by vaccination status. Figure S5: KM Plot Influenza NT, SGA, by vaccination status. Figure S6: KM Plot Influenza Qld, stillbirth, by vaccination status. Figure S7: KM Plot Pertussis Qld, preterm birth, by vaccination status. Figure S8: KM Plot Pertussis NT, preterm birth, by vaccination status. Figure S9: KM Plot Pertussis Qld, SGA, by vaccination status. Figure S10: KM Plot Pertussis NT, SGA, by vaccination status. Figure S11: KM Plot Pertussis Qld, Stillbirth, by vaccination status. [file BJO-133-1083-s002.docx]

**Supporting Information: Figures**


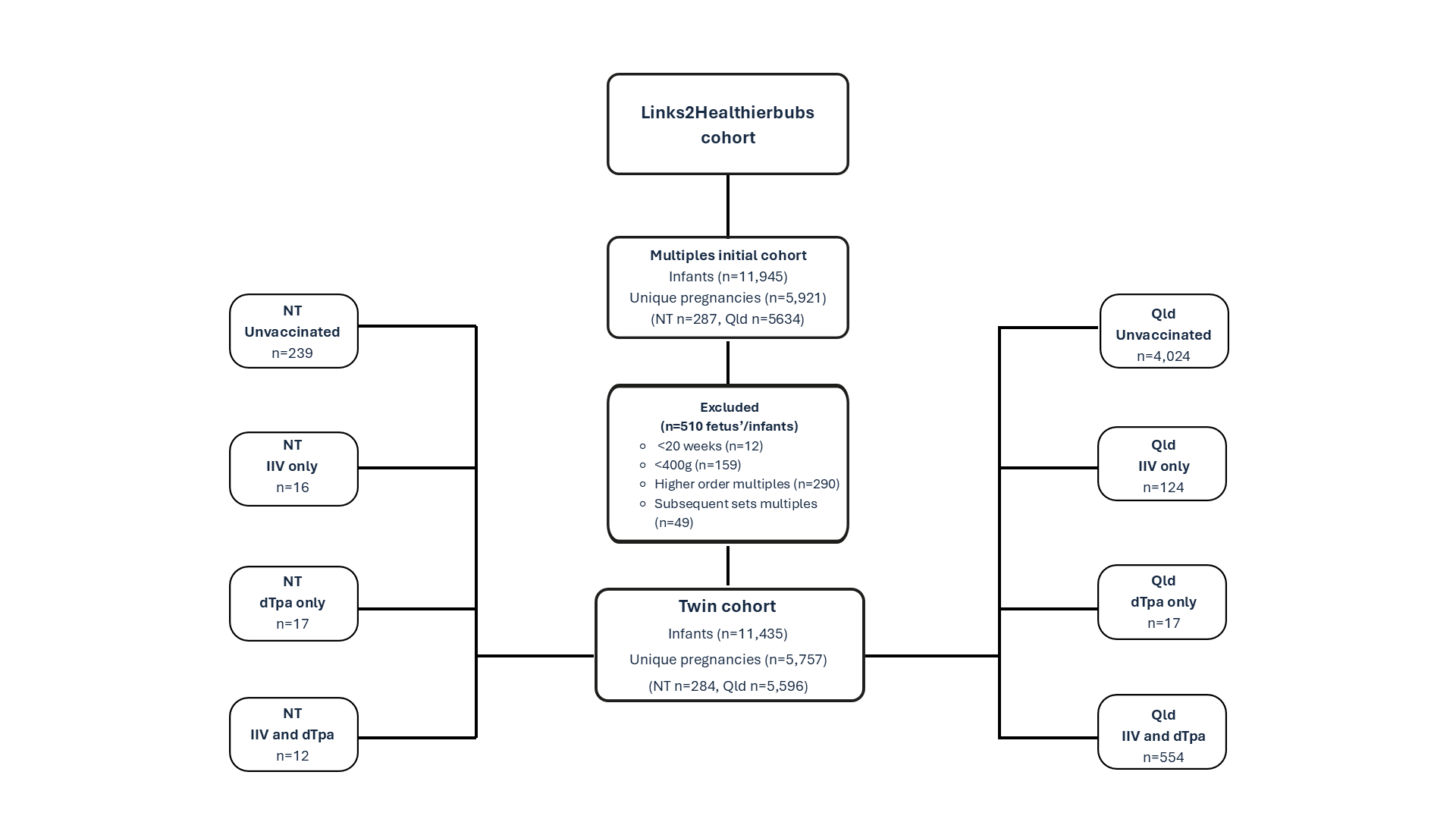


**Figure S1** Flow diagram of participants by jurisdiction and vaccination status (influenza and post- pertussis), 2012-2017


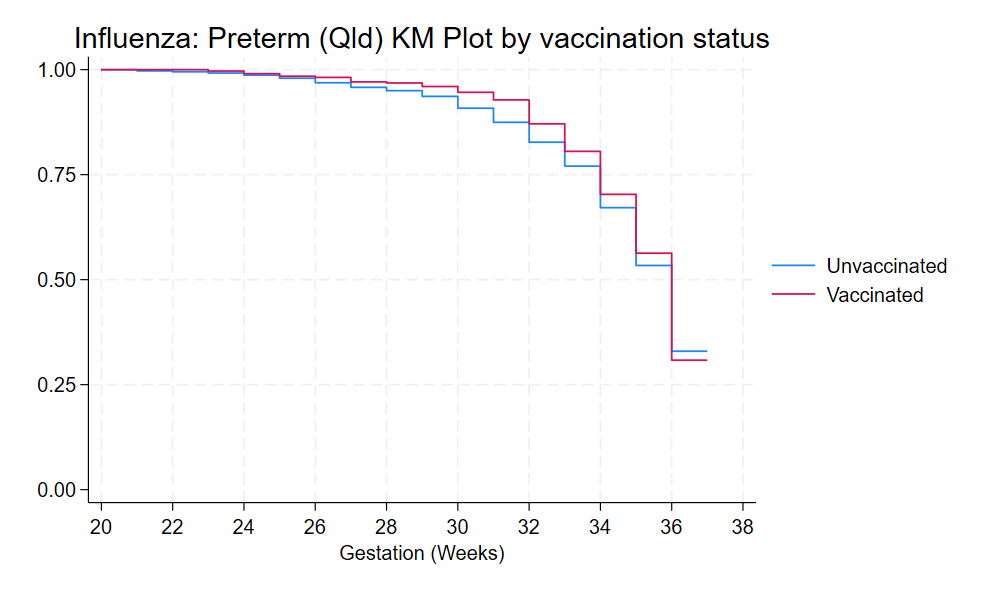

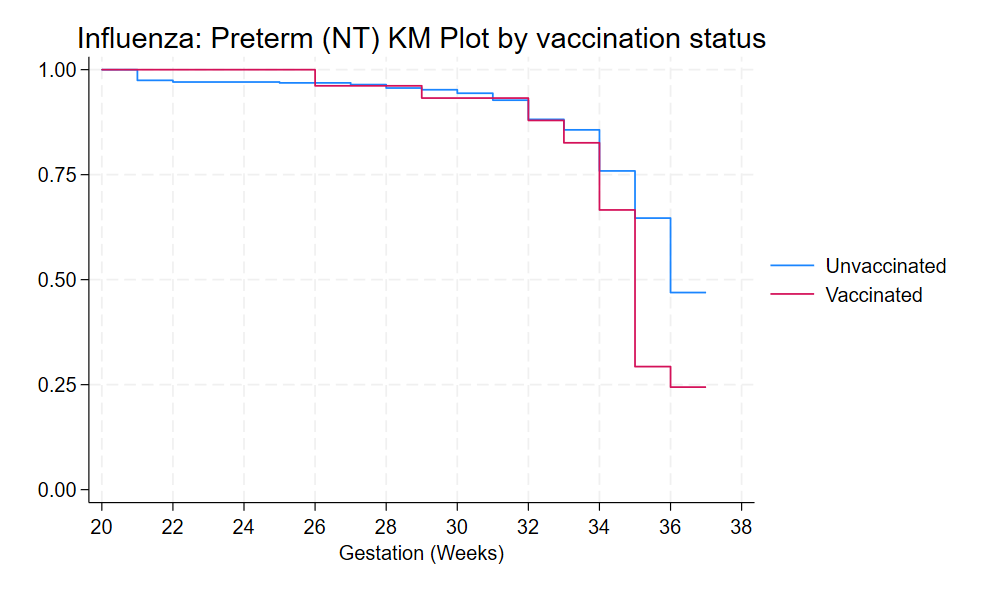


**Figure S2** KM Plot Influenza Qld, preterm birth, by vaccination status **Figure S3** KM Plot Influenza NT, preterm birth, by vaccination status


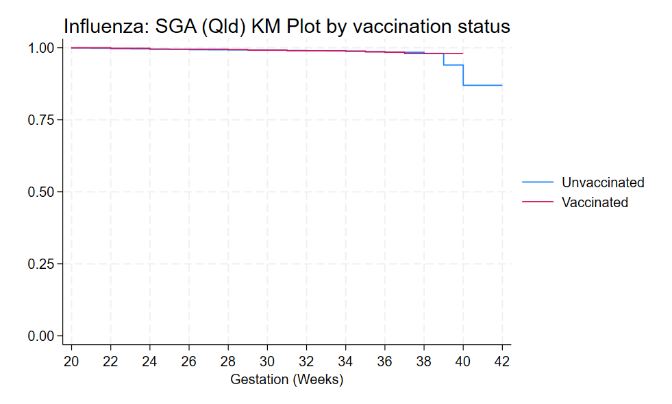

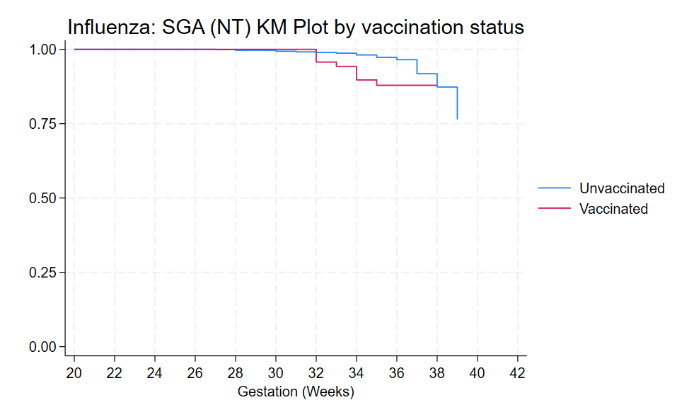


**Figure S4** KM Plot Influenza Qld, SGA, by vaccination status **Figure S5** KM Plot Influenza NT, SGA, by vaccination status


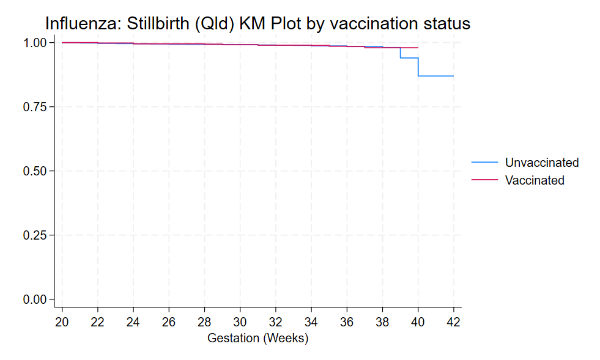


**Figure S6** KM Plot Influenza Qld, stillbirth, by vaccination status


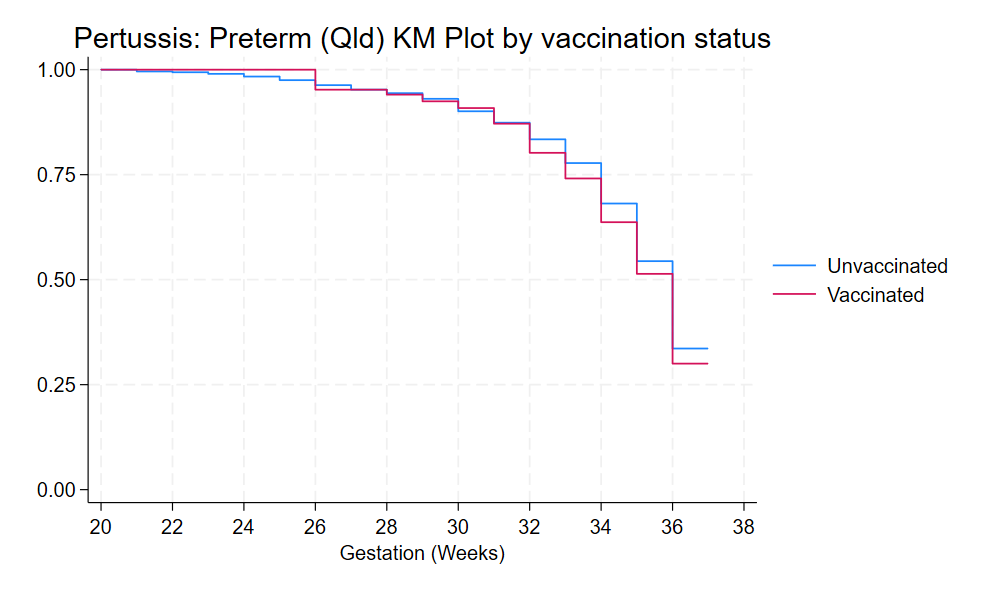

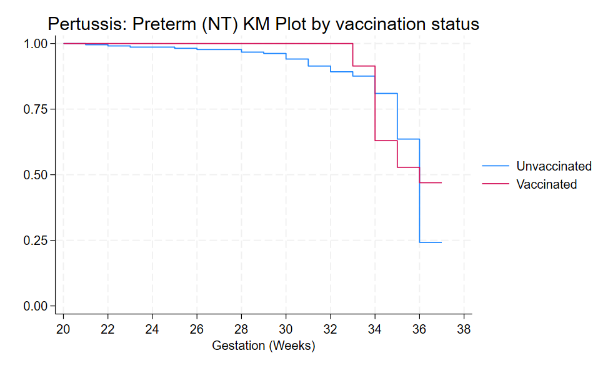


**Figure S7** KM Plot Pertussis Qld, preterm birth, by vaccination status **Figure S8** KM Plot Pertussis NT, preterm birth, by vaccination status


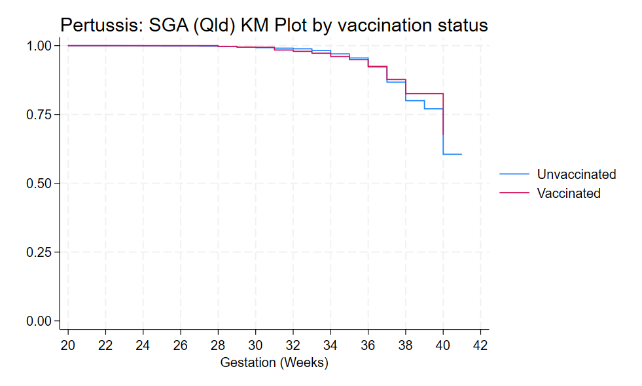

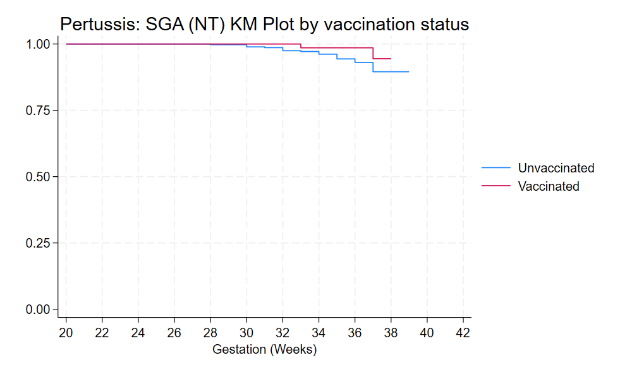


**Figure S9** KM Plot Pertussis Qld, SGA, by vaccination status **Figure S10** KM Plot Pertussis NT, SGA, by vaccination status


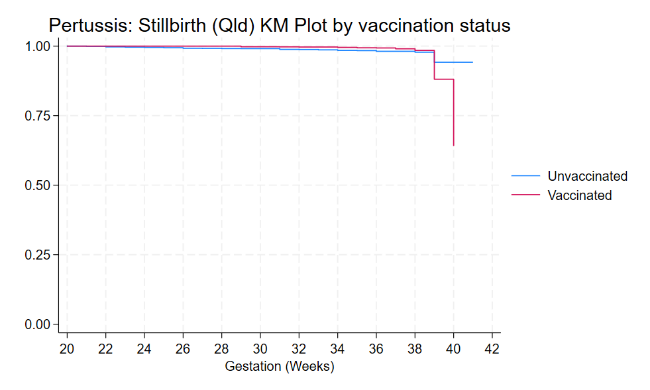


**Figure S11** KM Plot Pertussis Qld, Stillbirth, by vaccination status
