# Supplementary material for: The Risk of Adverse Birth Outcomes Among Twin Pregnancies After Influenza and Pertussis Vaccinations During Pregnancy: A Data Linkage Study
Source: BJOG. 2026 Jan 18;133(5):1083–92. doi: 10.1111/1471-0528.70156 (PMC12972857; doi:10.1111/1471-0528.70156)
Supplement: Supplementary file 2 — Table S1: Demographic, pregnancy and health characteristics—excluded pregnancies, multiple births 2012–2017. Table S2: Demographic, pregnancy and health characteristics, by jurisdiction, twin births 2012–2017. Table S3: Demographic, pregnancy and health characteristics unadjusted risk ratios, by jurisdiction and vaccination status, twin births influenza (2012) and post‐pertussis (Queensland 2014, Northern Territory 2015)—2017. Table S4: Comparison of crude and adjusted estimates using Cox‐proportional hazard and competing risks (Fine‐Grey) models with stillbirth a competing risk for preterm birth and small for gestational age. Table S5: Absolute risk of small for gestational age, stillbirth and preterm birth—overall, by jurisdiction and vaccination status. Table S6: Crude and adjusted Cox proportional hazard ratios for stillbirth and maternal influenza vaccination, Queensland twin births 2012–2017. Table S7: Crude and adjusted Cox proportional hazard ratios for small for gestational age and maternal influenza vaccination, Queensland and Northern Territory twin births 2012–2017. Table S8: Crude and adjusted Cox proportional hazard ratios for preterm birth and maternal influenza vaccination, Queensland and Northern Territory twin births 2012–2017. Table S9: Crude and adjusted Cox proportional hazard ratios for stillbirth and maternal pertussis vaccination, Queensland twin births 2014–2017. Table S10: Crude and adjusted Cox proportional hazard ratios for small for gestational age and maternal pertussis vaccination, Queensland (2014) and Northern Territory (2015)—2017 twin births. Table S11: Crude and adjusted Cox proportional hazard ratios for preterm birth and maternal pertussis vaccination, Queensland (2014) and Northern Territory (2015)—2017 twin births. Table S12: Sensitivity analyses to assess unmeasured confounding (E‐value). [file BJO-133-1083-s001.docx]

**Supporting Information: Tables**

**Table S1** Demographic, pregnancy and health characteristics – excluded pregnancies, multiple births 2012-2017

|  | **Triplet and Higher Order Multiples** | **Subsequent**  **Multiples** |
| --- | --- | --- |
| **Characteristic (N%)** | **n=98** | **n=25** |
| **Jurisdiction** |  |  |
| Northern Territory | NA | <5 (4) |
| Queensland | 98 (100) | 24 (96) |
| **Maternal age group at infant birth** |  |  |
| <20 years | NA | NA |
| 20-34 years | 60 (61) | 12 (48) |
| ≥35 years | 38 (39) | 13 (52) |
| First Nations | <5 (3) | <5 (16) |
| Australian born | 79 (81) | 21 (84) |
| Remote area of residence | NA | <5 (4) |
| Smoked during pregnancy | 7 (7) | <5 (16) |
| BMI in obese category (>30)^*†^ | 21 (24) | 5 (23) |
| **Multiple birth** |  |  |
| Triplet | 96 (97) | 25 (100) |
| Quadruplet | <5 (2) | NA |
| Quintuplet | <5 (1) | NA |
| **Pregnancy-based characteristics** |  |  |
| Antenatal care in 1st trimester | 78 (85) | 23 (92) |
| Public hospital birth^*^ | 53 (54) | 12 (50) |
| Primiparous | 50 (51) | NA |
| Caesarean section | 86 (89) | 19 (76) |
| Pregnancy-related condition^‡^ | 26 (27) | <5 (12) |
| Pre-existing medical condition^§^ | 21 (21) | 6 (24) |
| **Vaccination status** |  |  |
| Unvaccinated | 76 (78) | 16 (64) |
| Pertussis (dTpa) only | 12 (12) | 5 (20) |
| Pertussis and Influenza | 10 (10) | <5 (16) |
| **Birth outcomes** |  |  |
| Stillborn | 14 (5) | <5 (4) |
| SGA | 31 (11) | <5 (4) |
| Preterm | 95 (97) | 15 (60) |
| Moderate to Late Preterm | 58 (59) | 10 (40) |
| Very Preterm | 25 (26) | <5 (8) |
| Extremely Preterm | 12 (12) | <5 (12) |

* Qld data only

† Derived from body mass index variable using Australian categories.

‡ Derived variable: presence of one of more of gestational diabetes, gestational hypertension, preeclampsia, antepartum haemorrhage

§ Derived variable: presence of one of more of asthma, hypertension, diabetes (type I or II), anaemia, cardiac condition, renal condition

**Table S2** Demographic, pregnancy and health characteristics, by jurisdiction, twin births 2012-2017

|  | **Total Pregnancies** | **Queensland** | **Northern Territory** | **P-value**^§^ | |
| --- | --- | --- | --- | --- | --- |
| **Characteristic (N%)** | **n=5,757** | **n=5,474 (95)** | **n=283 (5)** |  |  |
| **Maternal age** |  |  |  |  |  |
| <20 years | 106 (2) | 94 (2) | 12 (4) |  |  |
| 20-34 years | 4,098 (71) | 3,887 (71) | 211 (75) | 0.001 |  |
| ≥35 years | 1,553 (27) | 1,493 (27) | 60 (21) |  |  |
| **Body Mass Index^*^** |  |  |  |  |  |
| Underweight | NA | 259 (5) | NA |  |  |
| Healthy | NA | 2,655 (50) | NA | NA |  |
| Overweight | NA | 1,298 (24) | NA |  |  |
| Obese | NA | 1,113 (21) | NA |  |  |
| **SEIFA Index Score** |  |  |  |  |  |
| Index 1-3 | 1,628 (28) | 1,541 (28) | 87 (31) |  |  |
| Index 4-6 | 1,634 (28) | 1,566 (29) | 68 (24) | 0.238 |  |
| Index 7-10 | 2,495 (43) | 2,367 (43) | 128 (45) |  |  |
| First Nations | 370 (6) | 286 (5) | 84 (30) | <0.001 |  |
| Australian born | 4,773 (83) | 4,533 (83) | 240 (85) | 0.398 |  |
| Remote area of residence | 217 (4) | 144 (3) | 73 (26) | <0.001 |  |
| **Parity** |  |  |  |  |  |
| Primiparous | 2,361 (41) | 2,262 (41) | 99 (35) |  |  |
| 1 prior birth | 1,831 (32) | 1,740 (32) | 91 (32) | 0.046 |  |
| ≥2 prior births | 1,565 (27) | 1,472 (27) | 93 (33) |  |  |
| **Pregnancy-based characteristics** |  |  |  |  |  |
| Antenatal care in 1st trimester | 4,396 (78) | 4,166 (78) | 230 (82) | 0.105 |  |
| Public hospital birth^*^ | NA | 2,937 (54) | NA | NA |  |
| Smoked during pregnancy | 668 (12) | 607 (11) | 61 (24) | <0.001 |  |
| Caesarean section | 4,053 (70) | 3,854 (70) | 199 (70) | 0.975 |  |
| Pregnancy-related condition^†^ | 1,117 (19) | 1,025 (19) | 92 (33) | <0.001 |  |
| Pre-existing medical condition^‡^ | 974 (17) | 890 (16) | 84 (30) | <0.001 |  |
| **Vaccination Status** |  |  |  |  |  |
| Unvaccinated | 4,171 (72) | 3,933 (72) | 238 (84) |  |  |
| Vaccinated | 1,586 (28) | 1,541 (28) | 45 (16) |  |  |
| Influenza only | 140 (2) | 124 (2) | 16 (6) |  |  |
| Pertussis only | 894 (16) | 877 (16) | 17 (6) |  |  |
| Influenza and Pertussis | 552 (10) | 540 (10) | 12 (5) |  |  |

Denominators reflect total number of unique pregnancies and differ due to missing data.

* Queensland data only

† Derived variable: presence of one of more of gestational diabetes, gestational hypertension, preeclampsia, antepartum haemorrhage

**‡** Derived variable: presence of one of more of asthma, hypertension, diabetes (type I or II), anaemia, cardiac condition, renal condition

§ Statistical difference between Qld and NT (measured by Χ^2^)

**Table S3** Demographic, pregnancy and health characteristics unadjusted risk ratios, by jurisdiction and vaccination status, twin births influenza (2012) and post-pertussis (Queensland 2014, Northern Territory 2015) - 2017

|  |  |  | **Queensland** | | **Northern Territory** | |
| --- | --- | --- | --- | --- | --- | --- |
|  | **Overall IIV** | **Overall  dTpa** | **IIV** | **Pertussis (post dTpa program >2014)** | **IIV** | **Pertussis (post dTpa program >2015)** |
| **Maternal age group at infant birth** |  |  |  |  |  |  |
| <20 years | 0.29 (0.11 - 0.78) | 0.38 (0.21 - 0.69) | 0.32 (0.12 - 0.87) | 0.34 (0.17 - 0.65) | NA | NA |
| 20-34 years | 1.03 (0.98 - 1.08) | 1.00 (0.96 - 1.04) | 1.02 (0.98 - 1.08) | 0.98 (0.94 - 1.03) | 1.11 (0.92 - 1.34) | 1.06 (0.83 - 1.35) |
| ≥35 years | 0.98 (0.86 - 1.12) | 1.06 (0.96 - 1.17) | 0.99 (0.86 - 1.13) | 1.12 (0.98 - 1.27) | 0.83 (0.36 - 1.90) | 0.89 (0.39 - 2.00) |
| **Demographics** |  |  |  |  |  |  |
| First Nations | 0.91 (0.67 - 1.25) | 0.65 (0.50 - 0.84) | 0.94 (0.66 - 1.35) | 0.54 (0.39 - 0.73) | 0.95 (0.52 - 1.77) | 0.44 (0.17 - 1.16) |
| Australian born | 0.97 (0.93 - 1.01) | 0.99 (0.96 - 1.02) | 0.97 (0.93 - 1.01) | 1.01 (0.98 - 1.05) | 0.97 (0.81 - 1.16) | 0.78 (0.60 - 1.01) |
| Remote area of residence | 0.91 (0.60 - 1.38) | 0.67 (0.48 - 0.94) | 0.84 (0.50 - 1.43) | 0.79 (0.49 - 1.28) | 1.28 (0.72 - 2.28) | 0.73 (0.33 - 1.60) |
| SEIFA Index 1 | 0.85 (0.67 - 1.08) | 0.70 (0.58 - 0.85) | 0.83 (0.64 - 1.07) | 0.61 (0.48 - 0.76) | 1.26 (0.67 - 2.35) | 0.83 (0.34 - 2.05) |
| Smoked during pregnancy | 0.66 (0.51 - 0.86) | 0.57 (0.47 - 0.69) | 0.66 (0.50 - 0.87) | 0.51 (0.41 - 0.64) | 0.77 (0.34 - 1.75) | 0.32 (0.08 - 1.31) |
| BMI obese category (>30)^*†^ | 0.91 (0.77 - 1.07) | 1.01 (0.90 - 1.14) | 0.91 (0.77 - 1.07) | 0.92 (0.80 - 1.07) | NA | NA |
| **Pregnancy-based characteristics** |  |  |  |  |  |  |
| Antenatal care in 1st trimester | 1.14 (1.10 - 1.18) | 1.14 (1.11 - 1.17) | 1.14 (1.10 - 1.18) | 1.12 (1.08 - 1.16) | 1.10 (0.96 - 1.27) | 1.24 (1.11 - 1.39) |
| Public hospital birth^*^ | 0.80 (0.73 - 0.88) | 0.91 (0.86 - 0.96) | 0.80 (0.73 - 0.88) | 0.79 (0.74 - 0.85) | NA | NA |
| Primiparous | 1.13 (1.03 - 1.23) | 1.17 (1.09 - 1.25) | 1.12 (1.03 - 1.23) | 1.37 (1.25 - 1.51) | 1.14 (0.70 - 1.86) | 1.88 (1.04 - 3.40) |
| Caesarean section | 1.00 (0.95 - 1.05) | 1.05 (1.01 - 1.09) | 1.00 ( 0.95 - 1.05) | 1.12 (1.07 - 1.19) | 1.07 (0.85 - 1.35) | 0.99 (0.75 - 1.32) |
| Pregnancy-related complication^‡^ | 1.14 (0.97 - 1.32) | 1.18 (1.05 - 1.33) | 1.19 (1.02 - 1.39) | 1.16 (1.00 - 1.35) | 0.52 (0.23 - 1.18) | 0.57 (0.27 - 1.23) |
| Pre-existing medical risk factor^§^ | 1.13 (0.95 - 1.33) | 1.19 (1.05 - 1.35) | 1.14 (0.96 - 1.36) | 0.95 (0.81 - 1.11) | 1.09 (0.62 - 1.93) | 0.89 (0.53 - 1.49) |

Pertussis vaccine (dTpa) in pregnancy was recommended from 2014 in Qld and 2015 in NT.

* Queensland data only

† Derived from body mass index variable using Australian categories

‡ Derived variable: presence of one of more of gestational diabetes, gestational hypertension, preeclampsia, antepartum haemorrhage

§ Derived variable: presence of one of more of asthma, hypertension, diabetes (type I or II), anaemia, cardiac condition, renal condition

**Table S4 Comparison of crude and adjusted estimates using Cox-proportional hazard and competing risks (Fine-Gray) models with stillbirth a competing risk for preterm birth and small for gestational age**

|  | **Cox proportional hazard**  **model** | | **Competing risks subdistribution hazard model** | | |
| --- | --- | --- | --- | --- | --- |
| **Influenza HR (95% CI) p-value HR_SD)_ (95% CI) p-value** | | | | | |
| **Queensland** | | | | | |
| SGA crude | 1.08 (0.87 - 1.35) | 0.478 | 1.09 (0.87 – 1.35) | 0.457 | |
| SGA adjusted | 1.10 (0.88 - 1.37) | 0.391 | 1.10 (0.89 – 1.37) | 0.370 | |
| Preterm birth crude | 1.05 (0.95 – 1.16) | 0.372 | 1.03 (0.96 – 1.12) | 0.475 | |
| Preterm birth adjusted*  (maternal age, parity, smoking, remoteness, season conception) | 1.05 (0.95 – 1.17) | 0.322 | 1.04 (0.95 – 1.13) | 0.381 | |
| **Northern Territory** | | |  | | |
| SGA crude | 1.80 (0.69 - 4.72) | 0.232 | 1.84 (0.71 – 4.75) | 0.208 | |
| SGA adjusted*  (maternal age, parity) | 1.85 (0.70 – 4.88) | 0.212 | 1.88 (0.73 – 4.86) | 0.191 | |
| Preterm birth crude | 1.26 (0.78 – 2.06) | 0.349 | 1.29 (0.80 – 2.07) | 0.299 | |
| Preterm birth adjusted | 1.18 (0.72 - 1.96) | 0.512 | 1.25 (0.76 – 2.05) | 0.389 | |
| **Pertussis** | | | | |  |
| **Queensland** | | | | | |
| SGA crude | 0.89 (0.73 - 1.08) | 0.230 | 0.91 (0.75 – 1.09) | 0.305 | |
| SGA adjusted*  (SEIFA, smoking, remoteness, parity) | 0.84 (0.69 – 1.02) | 0.074 | 0.86 (0.71 – 1.04) | 0.123 | |
| Preterm birth crude | 1.10 (1.01 - 1.20) | 0.028 | 1.11 (1.03 – 1.20) | 0.007 | |
| Preterm birth adjusted*  (maternal age, smoking, remoteness) | 1.12 (1.02 – 1.22) | 0.012 | 1.12 (1.04 – 1.21) | 0.002 | |
| **Northern Territory** |  |  |  |  | |
| SGA crude | 0.52 (0.17 - 1.60) | 0.254 | 0.53 (0.17 – 1.62) | 0.264 | |
| SGA adjusted | 0.50 (0.17 – 1.44) | 0.201 | 0.51 (0.18 – 1.45) | 0.205 | |
| Preterm birth crude | 0.83 (0.47 - 1.45) | 0.507 | 0.87 (0.52 – 1.45) | 0.593 | |
| Preterm birth adjusted | 0.78 (0.44 - 1.38) | 0.402 | 0.84 (0.49 – 1.43) | 0.521 | |

*Indicates the adjusted model includes confounders that differ to the model presented in the main analysis. The model shown represents a valid (proportional) model for the purpose of comparison between the HR and HR_SD_

**Table S5 Absolute risk of small for gestational age, stillbirth and preterm birth – overall, by jurisdiction and vaccination status**

|  | **Overall** | **Qld** | **NT** | | **Unvaccinated**  **Qld NT** | | **Vaccinated**  **Qld NT** | |
| --- | --- | --- | --- | --- | --- | --- | --- | --- |
| **Influenza** |  |  | |  |  | |  | |
| Small for gestational age | **8.2%** | **8.2%** | | **9.6%** | 8.1% | 9.0% | 8.4% | 14.6% |
| Stillbirth | **1.4%** | **1.4%** | | **1.6%** | 1.4% | 1.8% | 1.1% | 0.0% |
| Preterm birth | **66.3%** | **66.5%** | | **62.6%** | 66.4% | 62.5% | 67.1% | 63.6% |
| **Pertussis** |  |  | |  |  | |  | |
| Small for gestational age | 8.2% | 8.2% | | 9.6% | 8.60% | 10.0% | 7.0% | 6.3% |
| Stillbirth | 1.4% | 1.4% | | 1.6% | 1.6% | 1.8% | 0.7% | 0.0% |
| Preterm birth | 66.3% | 66.5% | | 62.6% | 67.4% | 63.5% | 64.0% | 56.3% |

**Table S6** Crude and adjusted Cox proportional hazard ratios for stillbirth and maternal influenza vaccination, Queensland twin births 2012-2017

| **Variable** | **Crude HR (95% CI)** | **p-value** | **Adjusted HR (95% CI)** | **p-value** |
| --- | --- | --- | --- | --- |
| **Maternal Vaccination Status** |  |  |  |  |
| Unvaccinated | REF |  | REF |  |
| Vaccinated (IIV) | 1.28 (0.68 - 2.40) | 0.447 | 1.28 (0.68 - 2.42) | 0.446 |
| **Maternal Age** |  |  |  |  |
| <20 years | 1.36 (0.43 – 4.31) | 0.604 | 1.02 (0.30 – 3.42) | 0.977 |
| 20-34 years | REF |  | REF |  |
| ≥35 years | 1.05 (0.69 – 1.60) | 0.826 | 1.17 (0.76 - 1.79) | 0.482 |
| **Remote** |  |  |  |  |
| Non-remote region | REF |  | REF |  |
| Remote region | 1.12 (0.41 - 3.05) | 0.820 | 1.04 (0.38 - 2.90) | 0.933 |
| **SEIFA Index*** |  |  |  |  |
| Index 1-3 | 1.67 (1.00 - 2.79) | 0.048 | 1.72 (1.02 - 2.89) | 0.043 |
| Index 4-6 | REF |  | REF |  |
| Index 7-10 | 1.14 (0.69 – 1.88) | 0.607 | 1.12 (0.68 – 1.84) | 0.663 |
| **Parity** | 0.92 (0.79 – 1.08) | 0.295 | 0.87 (0.74 – 1.01) | 0.068 |
| **Smoking Status** |  |  |  |  |
| Did not smoke | REF |  | REF |  |
| Smoked | 1.45 (0.84 - 2.49) | 0.183 | 1.50 (0.85 – 2.64) | 0.162 |
| **Season of Conception** |  |  |  |  |
| Spring | REF |  | REF |  |
| Summer | 0.92 (0.54 – 1.55) | 0.754 | 0.90 (0.53 - 1.53) | 0.696 |
| Autumn | 1.10 (0.66 – 1.83) | 0.722 | 1.08 (0.65 - 1.80) | 0.766 |
| Winter | 0.79 (0.46 – 1.34) | 0.375 | 0.80 (0.47 - 1.36) | 0.411 |

*SEIFA score based on the Australian Bureau of Statistics Index of Relative Socio-economic Advantage and Disadvantage (IRSAD) 2011

**Table S7** Crude and adjusted Cox proportional hazard ratios for small for gestational age and maternal influenza vaccination, Queensland and Northern Territory twin births 2012-2017

|  | **Queensland** | | | | | **Northern Territory** | | | |
| --- | --- | --- | --- | --- | --- | --- | --- | --- | --- |
| **Variable** | **Crude HR**  **(95% CI)** | **p-value** | | **Adjusted HR (95% CI)** | **p-value** | **Crude HR**  **(95% CI)** | **p-value** | **Adjusted HR ^†^**  **(95% CI)** | **p-value** |
| **Maternal Vaccination Status** |  |  |  | |  |  |  |  |  |
| Unvaccinated | REF |  | | REF |  | REF |  | REF |  |
| Vaccinated (IIV) | 1.08 (0.87 - 1.35) | 0.478 | | 1.10 (0.88 - 1.37) | 0.391 | 1.80 (0.69 - 4.72) | 0.232 | 1.33 (0.52 - 3.44) | 0.550 |
| **Maternal Age Category** |  |  | |  |  |  |  |  |  |
| <20 years | 2.00 (1.22 – 3.28) | 0.006 | | 1.27 (0.76 – 2.11) | 0.358 | 0.97 (0.14 – 6.57) | 0.975 | 0.68 (0.12 – 3.86) | 0.667 |
| 20-34 years | REF |  | | REF |  | REF |  | REF |  |
| ≥35 years | 1.12 (0.96 – 1.31) | 0.147 | | 1.30 (1.11 - 1.52) | 0.001 | 0.51 (0.23 - 1.15) | 0.103 | 1.62 (0.28 – 1.40) | 0.251 |
| **Remote residence** |  |  | |  |  |  |  |  |  |
| Non-remote region | REF |  | | REF |  | REF |  | REF |  |
| Remote region | 1.21 (0.75 – 1.93) | 0.437 | | 1.09 (0.68 - 1.75) | 0.725 | 2.83 (1.50 - 5.34) | 0.001 | 2.19 (1.03 – 4.66) | 0.041 |
| **SEIFA Index*** |  |  | |  |  |  |  |  |  |
| Index 1-3 | 1.19 (0.99 - 1.44) | 0.062 | | 1.19 (0.99 - 1.43) | 0.065 | 3.31 (1.40 - 7.85) | 0.007 | 2.41 (0.93 – 6.25) | 0.071 |
| Index 4-6 | REF |  | | REF |  | REF |  | REF |  |
| Index 7-10 | 0.97 (0.81 – 1.16) | 0.745 | | 0.96 (0.81 – 1.15) | 0.686 | 1.21 (0.49 – 2.99) | 0.671 | 1.16 (0.40 – 3.35) | 0.781 |
| **Parity** | 0.86 (0.80 – 0.93) | <0.001 | | 0.80 (0.74 – 0.86) | <0.001 | 1.13 (0.95 – 1.36) | 0.173 | 1.10 (0.91 – 1.33) | 0.321 |
| **Smoking Status** |  |  | |  |  |  |  |  |  |
| Did not smoke | REF |  | | REF |  | REF |  | REF |  |
| Smoked | 2.12 (1.76 - 2.55) | <0.001 | | 2.44 (2.01 – 2.96) | <0.001 | 2.68 (1.43 – 5.03) | 0.002 | 2.04 (1.14 - 3.67) | 0.017 |

*SEIFA score based on the Australian Bureau of Statistics Index of Relative Socio-economic Advantage and Disadvantage (IRSAD) 2011

**^†^**  Stratified by season of conception.

**Table S8** Crude and adjusted Cox proportional hazard ratios for preterm birth and maternal influenza vaccination, Queensland and Northern Territory twin births 2012-2017

|  | **Queensland** | | | | **Northern Territory** | | | |  |
| --- | --- | --- | --- | --- | --- | --- | --- | --- | --- |
| **Variable** | **Crude HR**  **(95% CI)** | **p-value** | **Adjusted HR ^†^**  **(95% CI)** | **p-value** | **Crude HR**  **(95% CI)** | **p-value** | **Adjusted HR (95% CI)** | **p-value** | |
| **Maternal Vaccination Status** |  |  |  |  |  |  |  |  | |
| Unvaccinated | REF |  | REF |  | REF |  | REF |  | |
| Vaccinated (IIV) | 1.05 (0.95 – 1.15) | 0.347 | 0.93 (0.84 - 1.03) | 0.184 | 1.26 (0.78 – 2.06) | 0.349 | 1.18 (0.72 - 1.96) | 0.512 | |
| **Maternal Age** |  |  |  |  |  |  |  |  | |
| <20 years | 1.55 (1.23 – 1.95) | <0.001 | 1.06 (0.84 – 1.34) | 0.622 | 1.47 (0.77 – 2.80) | 0.240 | 1.24 (0.61 – 2.51) | 0.556 | |
| 20-34 years | REF |  | REF |  | REF |  | REF |  | |
| ≥35 years | 1.00 (0.93 – 1.08) | 0.995 | 1.03 (0.95 - 1.11) | 0.446 | 0.75 (0.51 - 1.10) | 0.144 | 0.80 (0.53 – 1.21) | 0.286 | |
| **Remote** |  |  |  |  |  |  |  |  | |
| Non-remote region | REF |  | REF |  | REF |  | REF |  | |
| Remote region | 1.24 (1.02 – 1.51) | 0.034 | 1.18 (0.97 - 1.44) | 0.106 | 1.62 (1.17 - 2.24) | 0.003 | 1.19 (0.74 - 1.90) | 0.479 | |
| **SEIFA Index*** |  |  |  |  |  |  |  |  | |
| Index 1-3 | 0.98 (0.90 – 1.07) | 0.726 | 1.01 (0.93 - 1.11) | 0.752 | 1.94 (1.28 - 2.95) | 0.002 | 1.63 (0.97 – 2.72) | 0.063 | |
| Index 4-6 | REF |  | REF |  | REF |  | REF |  | |
| Index 7-10 | 0.95 (0.88 – 1.02) | 0.168 | 0.96 (0.89 – 1.04) | 0.330 | 1.24 (0.82 – 1.85) | 0.306 | 1.26 (0.84 – 1.90) | 0.271 | |
| **Parity** | 0.92 (0.90 – 0.95) | <0.001 | 0.98 (0.95 – 1.00) | 0.099 | 1.04 (0.94 – 1.16) | 0.431 | 1.05 (0.94 – 1.18) | 0.364 | |
| **Smoking Status** |  |  |  |  |  |  |  |  | |
| Did not smoke | REF |  | REF |  | REF |  | REF |  | |
| Smoked | 1.19 (1.08 - 1.32) | 0.001 | 1.13 (1.01 - 1.26) | 0.029 | 1.31 (0.93 - 1.86) | 0.121 | 1.14 (0.79 - 1.63) | 0.487 | |
| **Season of Conception** |  |  |  |  |  |  |  |  | |
| Spring | REF |  | REF |  | NA | NA | NA | NA | |
| Summer | 1.01 (0.92 – 1.11) | 0.832 | 1.01 (0.92 – 1.11) | 0.874 | 1.14 (0.75 – 1.73) | 0.534 | 1.19 (0.77 - 1.82) | 0.430 | |
| Autumn | 1.03 (0.94 – 1.13) | 0.466 | 1.00 (0.91 – 1.10) | 0.978 | 1.20 (0.79 – 1.83) | 0.389 | 1.04 (0.68 – 1.61) | 0.849 | |
| Winter | 1.04 (0.95 – 1.14) | 0.401 | 1.01 (0.92 – 1.10) | 0.875 | 1.13 (0.75 – 1.71) | 0.567 | 1.09 (0.72 – 1.65) | 0.697 | |

*SEIFA score based on the Australian Bureau of Statistics Index of Relative Socio-economic Advantage and Disadvantage (IRSAD) 2011

**^†^** Stratified by preterm birth category

**Table S9** Crude and adjusted Cox proportional hazard ratios for stillbirth and maternal pertussis vaccination, Queensland twin births 2014 - 2017

| **Variable** | **Crude HR (95% CI)** | **p-value** | **Adjusted HR (95% CI)** | **p-value** |
| --- | --- | --- | --- | --- |
| **Maternal Vaccination Status** |  |  |  |  |
| Unvaccinated | REF |  | REF |  |
| Vaccinated (dTpa) | 1.15 (0.61 - 2.18) | 0.660 | 1.14 (0.60 - 2.15) | 0.691 |
| **Maternal Age** |  |  |  |  |
| <20 years | 1.35 (0.33 – 5.56) | 0.676 | 1.00 (0.23 – 4.34) | 0.995 |
| 20-34 years | REF |  | REF |  |
| ≥35 years | 1.21 (0.73 – 2.00) | 0.456 | 1.34 (0.80 – 2.23) | 0.262 |
| **Remote** |  |  |  |  |
| Non-remote region | REF |  | REF |  |
| Remote region | 1.72 (0.63 - 4.71) | 0.290 | 1.68 (0.59 – 4.77) | 0.333 |
| **SEIFA Index*** |  |  |  |  |
| Index 1-3 | 1.33 (0.72 - 2.46) | 0.366 | 1.35 (0.71 - 2.58) | 0.356 |
| Index 4-6 | REF |  | REF |  |
| Index 7-10 | 1.01 (0.56 – 1.83) | 0.970 | 1.01 (0.55 – 1.85) | 0.981 |
| **Parity** | 0.94 (0.79 – 1.12) | 0.473 | 0.89 (0.76 – 1.06) | 0.200 |
| **Smoking Status** |  |  |  |  |
| Did not smoke | REF |  | REF |  |
| Smoked | 1.48 (0.76 - 2.90) | 0.252 | 1.52 (0.75 - 3.08) | 0.245 |

*SEIFA score based on the Australian Bureau of Statistics Index of Relative Socio-economic Advantage and Disadvantage (IRSAD) 2011

**Table S10** Crude and adjusted Cox proportional hazard ratios for small for gestational age and maternal pertussis vaccination, Queensland (2014) and Northern Territory (2015) – 2017 twin births*

|  | **Queensland** | | | | **Northern Territory** | | | | | |  |
| --- | --- | --- | --- | --- | --- | --- | --- | --- | --- | --- | --- |
| **Variable** | **Crude HR**  **(95% CI)** | **p-value** | **Adjusted HR^†^**  **(95% CI)** | **p-value** | **Crude HR**  **(95% CI)** | **p-value** | **Adjusted HR**  **(95% CI)** | | **p-value** | | |
| **Maternal Vaccination Status** |  |  |  |  |  |  |  | |  | |  |
| Unvaccinated | REF |  | REF |  | REF |  | REF | |  | |  |
| Vaccinated (dTpa) | 0.89 (0.73 - 1.08) | 0.230 | 0.85 (0.70 - 1.03) | 0.090 | 0.52 (0.17 - 1.60) | 0.254 | 0.50 (0.17 - 1.44) | | 0.201 | |  |
| **Maternal Age** |  |  |  |  |  |  |  | |  | |  |
| <20 years | 2.71 (1.61 – 4.58) | <0.001 | 1.41 (0.80 – 2.49) | 0.238 | NA |  | NA | |  | |  |
| 20-34 years | REF |  | REF |  | REF |  | REF | |  | |  |
| ≥35 years | 1.10 (0.90 – 1.34) | 0.361 | 1.17 (0.96 - 1.42) | 0.126 | 0.32 (0.10 – 1.07) | 0.064 | 0.37 (0.11 – 1.20) | | 0.098 | |  |
| **Remote** |  |  |  |  |  |  |  | |  | |  |
| Non-remote region | REF |  | REF |  | REF |  | REF | |  | |  |
| Remote region | 1.42 (0.81 - 2.50) | 0.225 | 1.42 (0.80 - 2.53) | 0.231 | 3.54 (1.59 – 7.88) | 0.002 | 1.40 (0.37 - 5.39) | | 0.621 | |  |
| **SEIFA Index^†^** |  |  |  |  |  |  |  | |  | |  |
| Index 1-3 | 1.17 (0.92 - 1.48) | 0.201 | 1.09 (0.86 - 1.38) | 0.482 | 5.52 (1.57 – 19.38) | 0.008 | 3.58 (0.71 - 18.14) | | 0.124 | |  |
| Index 4-6 | REF |  | REF |  | REF |  | REF |  | |  |  |
| Index 7-10 | 1.10 (0.88 – 1.37) | 0.403 | 1.08 (0.87 – 1.35) | 0.476 | 1.97 (0.55 – 7.02) | 0.294 | 2.03 (0.54 – 7.56) | | 0.292 | |  |
| **Parity** | NA |  | NA |  | 1.18 (0.96 – 1.46) | 0.124 | 1.19 (0.94 – 1.49) | | 0.143 | |  |
| **Smoking Status** |  |  |  |  |  |  |  | |  | |  |
| Did not smoke | REF |  | REF |  | REF |  | REF | |  | |  |
| Smoked | 2.18 (1.73 - 2.75) | <0.001 | 2.33 (1.82 - 2.97) | <0.001 | 4.16 (1.85 – 9.36) | 0.001 | 2.32 (0.99 - 5.43) | | 0.052 | |  |

* Pertussis (dTpa) recommended during pregnancy from 2014 in Qld and 2015 in NT

**^†^** SEIFA score based on the Australian Bureau of Statistics Index of Relative Socio-economic Advantage and Disadvantage (IRSAD) 2011

^‡^ Qld stratified by parity (0, 1, ≥2 prior births)

**Table S11** Crude and adjusted Cox proportional hazard ratios for preterm birth and maternal pertussis vaccination, Queensland (2014) and Northern Territory (2015) – 2017 twin births*

|  | **Queensland** | | | | **Northern Territory** | | | |
| --- | --- | --- | --- | --- | --- | --- | --- | --- |
| **Variable** | **Crude HR**  **(95% CI)** | **p-value** | **Adjusted HR^†^**  **(95% CI)** | **p-value** | **Crude HR**  **(95% CI)** | **p-value** | **Adjusted HR**  **(95% CI)** | **p-value** |
| **Maternal Vaccination Status** |  |  |  |  |  |  |  |  |
| Unvaccinated | REF |  | REF |  | REF |  | REF |  |
| Vaccinated (dTpa) | 1.10 (1.01 – 1.20) | 0.028 | 1.02 (0.93 - 1.11) | 0.656 | 0.83 (0.47 - 1.45) | 0.507 | 0.78 (0.44 - 1.38) | 0.402 |
| **Maternal Age** |  |  |  |  |  |  |  |  |
| <20 years | 1.38 (1.04 – 1.84) | 2.24 | 1.02 (0.76 – 0.36) | 0.888 | 1.41 (0.51 – 3.85) | 0.505 | 0.71 (0.24 – 2.13) | 0.543 |
| 20-34 years | REF |  | REF |  | REF |  | REF |  |
| ≥35 years | 0.96 (0.87 - 1.05) | 0.340 | 1.03 (0.93 - 1.13) | 0.586 | 0.47 (0.27 – 0.84) | 0.010 | 0.51 (0.28 – 0.93) | 0.029 |
| **Remote** |  |  |  |  |  |  |  |  |
| Non-remote region | REF |  | REF |  | REF |  | REF |  |
| Remote region | 1.28 (1.01 - 1.63) | 0.045 | 1.22 (0.96 - 1.56) | 0.110 | 2.01 (1.31 - 3.07) | 0.001 | 1.71 (0.89 - 3.31) | 0.109 |
| **SEIFA Index**^‡^ |  |  |  |  |  |  |  |  |
| Index 1-3 | 0.98 (0.88 - 1.10) | 0.780 | 1.00 (0.90 - 1.12) | 0.948 | 2.43 (1.37 – 4.33) | 0.002 | 1.53 (0.73 – 3.23) | 0.260 |
| Index 4-6 | REF |  | REF |  | REF |  | REF |  |
| Index 7-10 | 0.92 (0.84 – 1.02) | 0.112 | 0.93 (0.84 – 1.02) | 0.133 | 1.38 (0.78 – 2.43) | 0.267 | 1.24 (0.70 – 2.21) | 0.460 |
| **Parity** | 0.91 (0.88 – 0.94) | <0.001 | 0.95 (0.92 – 0.99) | 0.008 | 0.96 (0.84 – 1.11) | 0.622 | 0.98 (0.85 – 1.14) | 0.816 |
| **Smoking Status** |  |  |  |  |  |  |  |  |
| Did not smoke | REF |  | REF |  | REF |  | REF |  |
| Smoked | 1.15 (1.02 - 1.31) | 0.026 | 1.11 (0.97 - 1.27) | 0.136 | 1.16 (0.70 - 1.91) | 0.560 | 0.82 (0.47 - 1.42) | 0.468 |

*Pertussis (dTpa) recommended during pregnancy from 2014 in Qld and 2015 in NT

^†^ Stratified by preterm birth category

^‡^ SEIFA score based on the Australian Bureau of Statistics Index of Relative Socio-economic Advantage and Disadvantage (IRSAD) 2011

**Table S12 Sensitivity analyses to assess unmeasured confounding (E-value)**

|  | **Adjusted HR**  **(95% CI)** | **p-value** | **E-value point estimate** |
| --- | --- | --- | --- |
| **INFLUENZA** |  |  |  |
| **Queensland** |  |  |  |
| Stillbirth | 1.28 (0.68 - 2.42) | 0.446 | 1.88 |
| SGA | 1.10 (0.88 - 1.37) | 0.391 | 1.43 |
| Preterm birth | 0.93 (0.84 - 1.03) | 0.184 | 1.36 |
| **Northern Territory** |  |  |  |
| Stillbirth | NA | NA | NA |
| SGA | 1.33 (0.52 - 3.44) | 0.550 | 1.99 |
| Preterm birth | 1.18 (0.72 - 1.96) | 0.512 | 1.64 |
| **PERTUSSIS** |  |  |  |
| **Queensland** |  |  |  |
| Stillbirth | 1.14 (0.60 - 2.15) | 0.691 | 1.54 |
| SGA | 0.85 (0.70 - 1.03) | 0.090 | 1.63 |
| Preterm birth | 1.02 (0.93 - 1.11) | 0.656 | 1.16 |
| **Northern Territory** |  |  |  |
| Stillbirth | NA | NA | NA |
| SGA | 0.50 (0.17 - 1.44) | 0.201 | 3.41 |
| Preterm birth | 0.78 (0.44 - 1.38) | 0.402 | 1.88 |
